# The Effect of Crystal Defects on 3D High-Resolution Diffraction Peaks: A FFT-Based Method

**DOI:** 10.3390/ma11091669

**Published:** 2018-09-09

**Authors:** Komlavi Senyo Eloh, Alain Jacques, Gabor Ribarik, Stéphane Berbenni

**Affiliations:** 1 Laboratory of Excellence on Design of Alloy Metals for low-mAss Structures (DAMAS), Université de Lorraine, f-57073 Metz, France; komlavi-senyo.eloh@univ-lorraine.fr (K.S.E.); ribarik@elte.hu (G.R.); stephane.berbenni@univ-lorraine.fr (S.B.); 2 Institut Jean Lamour (IJL), Université de Lorraine, CNRS, f-54000 Nancy, France; 3 Laboratoire d’Etude des Microstructures et de Mécanique des Matériaux (LEM3), Université de Lorraine, CNRS, f-57073 Metz, France; 4 Department of Materials Physics, Eötvös University, h-1117 Budapest, Hungary

**Keywords:** dislocations, diffraction, fast Fourier transform (FFT)-based method, discrete green operator, voxelization artifacts, sub-voxel method, simulated diffraction peaks, scattered intensity

## Abstract

Forward modeling of diffraction peaks is a potential way to compare the results of theoretical mechanical simulations and experimental X-ray diffraction (XRD) data recorded during in situ experiments. As the input data are the strain or displacement field within a representative volume of the material containing dislocations, a computer-aided efficient and accurate method to generate these fields is necessary. With this aim, a current and promising numerical method is based on the use of the fast Fourier transform (FFT)-based method. However, classic FFT-based methods present some numerical artifacts due to the Gibbs phenomenon or “aliasing” and to “voxelization” effects. Here, we propose several improvements: first, a consistent discrete Green operator to remove “aliasing” effects; and second, a method to minimize the voxelization artifacts generated by dislocation loops inclined with respect to the computational grid. Then, we show the effect of these improvements on theoretical diffraction peaks.

## 1. Introduction

X-ray diffraction (XRD) is one of the most powerful non-destructive tools to investigate materials, as their wavelength is commensurate with the distance between atoms within a crystal [1,2,3,4,5,6,7,8,9]. Successive improvements of both the X-ray sources (from X-ray tubes to third generation synchrotrons) and detectors (from photographic plates and gas counters to fast two-dimensional arrays) have led to a tremendous increase in the quantity of data recorded per unit time, allowing real time in situ or in operando measurements [10,11]. It is now possible to determine the 3D grain microstructure of a bulk material with a submicron resolution (using topo-tomography), to follow the evolution of the elastic strain state of the grains of a polycrystal during mechanical tests (3D-XRD, far field diffractometry), or to measure the distribution of strains within a few grains in real time (2D diffractometry) [12,13]. Such experiments result in terabytes of data recorded within a few days, which need to be analyzed efficiently. In fact, only a low fraction of those data is actually treated because scientists lack both time and numerical tools (software) for further analysis [14]. 

The classical techniques used to analyze the 1D or 2D diffraction patterns recorded during tests performed on polycrystalline specimens such as the Rietveld method, the square sines method to measure internal stresses, or CMWP (convolutional multiple whole profile) fitting for dislocations content often rely on simplified and mathematically tractable models of a microstructure. Calculations which may involve a simplifying hypothesis lead to a general formula which can be used to fit one or several parameters of the microstructure (dislocation densities and type, internal stress tensor etc.) to the diffraction pattern (peak profiles, variation of the 2*θ*_B_ angle with orientation etc.).

During the last 10 years, several authors proposed the opposite approach: forward modeling [15,16,17,18,19,20,21,22]. This requires the design of a microstructure and the simulation of its behavior (often under process or thermo-mechanical solicitation), and the computation of the elastic strain field or the displacement field. The last step is the generation through a ‘virtual diffractometer’ of a theoretical diffraction pattern (different ***G*** vectors and different orientations of the lattice planes), which can be compared with the experimental one. Depending on the size of the simulated representative volume of matter and the experimental conditions such as the X-ray beam coherence, different assumptions can be made such as a coherent beam (where the amplitudes scattered by different points add) or an incoherent beam (scattered intensities add), or for a partially coherent beam where a full calculation may be necessary. Such modeling can be quite successful and can be used to validate the different steps involved, mainly the microstructure and the constitutive law used to simulate the material’s behavior.

However, as diffraction peaks contain information on different scales of a specimen: from average quantities such as Type I (average) stresses related to the peaks’ positions, Type II (at grain level) stresses related to its width, and Type III stresses (near the core of defects such as dislocations) related to the peaks’ tails, a realistic simulation of a diffraction peak requires a description of a material’s representative volume element with a very fine mesh, i.e., a huge amount of CPU time with classical methods used for simulations such as the finite element method. 

Numerical approaches based on the fast Fourier transform (FFT) for calculating the stress and strain fields within a composite material received a surge of interest since the pioneering work of Moulinec and Suquet [23,24]. They were first developed to compute effective properties and mechanical field of linear elastic composites [23,24,25,26] and were extended to heterogeneous materials with eigenstrains (dislocations, thermal strains etc.) [14,27,28,29,30]. They are also used for conductivity problems [31], non-linear materials [25,27], and viscoplastic or elasto-viscoplastic polycrystals [32,33,34,35,36]. Today, FFT-based approaches represent an attractive alternative to the finite element method because of lower computation time [32].

However initial tests indicate that the displacement field computed (essential for diffraction pattern generation) with FFT algorithms presents some numerical artifacts. These numerical artifacts are due to Gibbs phenomenon or “aliasing” and to voxelization. The accuracy of the calculated strain or displacement field is strongly influenced by these shortcomings and the simulated peaks may provide wrong information on mechanical behavior or material characteristics. Therefore, it is important to control these artifacts in order to simulate correct diffraction pattern in the case of a microstructure containing different phases, grains, and crystal defects. 

The aim of this paper is to improve the accuracy of the displacement field for diffraction peak generation. This improvement is based on the introduction of a consistent discrete periodized Green operator associated with the displacement field in order to take explicitly into account the discreteness of the discrete Fourier transform method [37]. The improvement of the voxelization in FFT-method is performed through a sub-voxelization method described for inclined dislocation loops. These improvements are reported and discussed in the present contribution. In the Section 2, the FFT-based method to compute the displacement field in a periodic medium is described. In Section 3, the treatment of voxelization problems in FFT-based approaches by a sub-voxelization method is detailed in the case of slip plane not conforming to FFT grid. In the Section 4, simulation of diffraction peaks is reported and discussed.

## 2. Fast Fourier Transform (FFT)-Based Numerical Calculation of the Displacement Field and Periodized Green Operators

### 2.1. FFT-Based Algorithm and Mechanical Fields

Let us consider a homogeneous elastic medium with eigenstrain assuming a periodic unit cell discretized in N×N×N voxels and subjected to a uniform overall strain tensor denoted E. Here, this overall strain is the spatial average of the strain field in the unit cell (with external loading and a given eigenstrain field). The unit cell may contain voxel size defects (0D) such as chemical inhomogeneities, line defects (1D) with arbitrary shape and distribution, planar (2D) defects such as stacking faults, or 3D precipitates which are all modeled with an eigenstrain tensor. (See [14].) These defects create a displacement field and thus generate strain and stress fields [38].

The displacement vector is denoted u and in the forthcoming equations, x denotes any position vector within the unit cell. All vector and tensor fields will be written using bold characters.

Starting from the equation for mechanical equilibrium, div σ(x)=0, and using field equations (strain compatibility, generalized Hooke’s law, decomposition of total strain compatible strain into elastic strain and eigenstrain), the displacement field is given at every position by the Green’s function technique [39]:

(1) $$\;u\left(x\right)=\left(B*c^{0}:\varepsilon^{*}\right)\left(x\right)\;$$

where the symbol ∗ denotes the spatial convolution product, $c^{0}$ is the homogeneous linear elastic stiffness, $\varepsilon^{*}$ is the eigenstrain field and B is a third order Green operator defined in Fourier space as:

(2) $$\;\hat{B_{ijk}}\left(\xi \right)=\frac{i}{2}\left(\hat{G_{ij}}\xi_{k}+\hat{G_{ik}}\xi_{j}\right)\;$$

in which $\hat{B}$ is the Fourier transform of B and $\hat{G}$ is the Fourier transform of the elastic Green tensor [39]. Therefore, using the Fourier transform of spatial convolution product, Equation (1) can be written in Fourier space as:

(3) $$\;\hat{u}\left(\xi \right)=\;\hat{B}\left(\xi \right):c^{0}:\hat{\varepsilon^{*}}\left(\xi \right)\;$$

Several numerical results showed that the use of the third order operator $\hat{B}$ derived from the classic Green $\hat{G}$ leads to spurious oscillations on the computed displacement field near materials discontinuities and dislocations [37]. The discrete Fourier transform (DFT) used in this algorithm indeed transforms a periodic function in real space into a periodic function in reciprocal space. However, the operator $\hat{B}$ commonly used is the continuous analytic operator truncated to the size of the unit cell of the reciprocal space: it is not periodic function. To fix this problem, we very recently developed a periodized consistent discrete Green operator using the DFT. The mathematical derivations of this discrete Green operator are given elsewhere [37]. The Fourier transform of this discrete Green operator denoted $\hat{B{}^{\prime }}$ is written as function of $\hat{B}$ and reads:

$$\;{\hat{B}}^{\prime }\left(\xi_{ijk}\right)=A_{ijk}\overset{+\infty }{\underset{m,n,p=-\infty }{\sum }}\frac{{\left(-1\right)}^{m+n+p}}{\left(mN+i\right)}\frac{1}{\left(nN+j\right)}\frac{1}{\left(pN+k\right)}\hat{B}\left(\xi_{mN+i,nN+j,pN+k}\right)\;\;$$

(4) $$\mathrm{With}\;A_{ijk}={\left(\frac{N}{\pi }\right)}^{3}\sin\left(\frac{i\pi }{N}\right)\sin\left(\frac{j\pi }{N}\right)\sin\left(\frac{k\pi }{N}\right)\;$$

Discrete frequencies appearing in this equation are given when *N* is even by (*T* is the period of the unit cell):

$$\;\xi =\left(-\frac{N}{2}+1\right)\frac{1}{T},\left(-\frac{N}{2}+2\right)\frac{1}{T},\ldots ,-\frac{1}{T},0,\frac{1}{T},\ldots .\left(\frac{N}{2}-1\right)\frac{1}{T},\left(\frac{N}{2}\right)\frac{1}{T}\;$$

Here, the sum on the $\hat{B}$ operator is extended to the whole reciprocal space (in practice for m,n,p up to a few tens) and folded up onto the unit cell of the DFT with suitable coefficients. The inverse transform of $\hat{u}\left(\xi \right)$ gives the displacement field at the center of each voxel.

We can also compute the displacement field at each voxel’s corner with a shifted operator using the shift theorem:

(5) $$\;{\hat{B{}^{\prime }}}^{\prime }\left(\xi_{ijk}\right)=A_{ijk}e^{p\pi \frac{i+j+k}{N}}\overset{+\infty }{\underset{m,n,p=-\infty }{\sum }}\frac{1}{\left(mN+i\right)}\frac{1}{\left(nN+j\right)}\frac{1}{\left(pN+k\right)}\hat{B}\left(\xi_{mN+i,nN+j,pN+k}\right)\;\;$$

### 2.2. Numerical Examples

**Figure 1:**
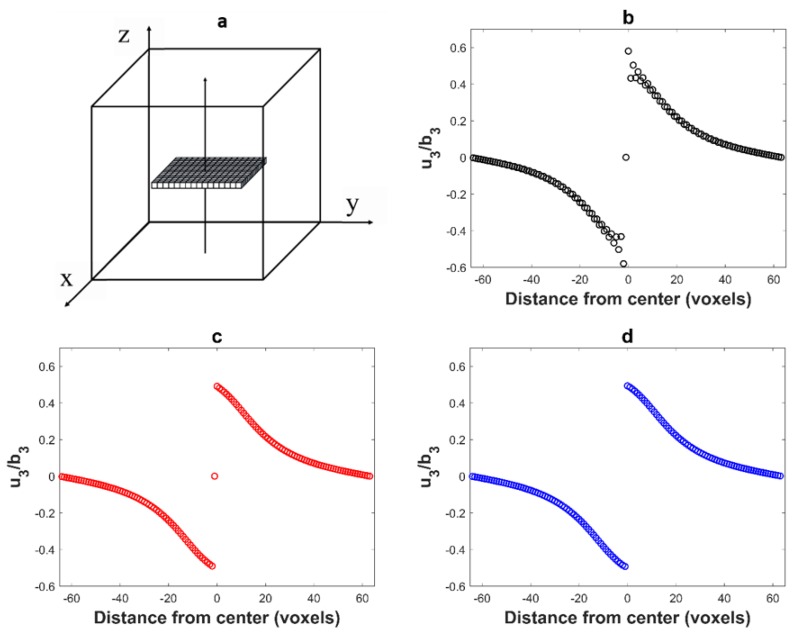
(**a**) Simulation of a square dislocation loop in plane (001) by a platelet with eigenstrain; (**b**) component $u_{3}$ of the displacement field (normalized by $b_{3}$) along the *z* axis (arrow) computed with the Green operator B and showing spurious oscillations; (**c**) same component $u_{3}$ computed with $B^{\prime }$. The displacement at voxel (64,64,64) is zero in the center of the inclusion (**c**) and $b_{3}/2$ on its surface; (**d**) same component $u_{3}$ computed with $B^{\prime \prime }$.

Let us consider a homogeneous material with isotropic elastic constants: Young’s modulus E=333.4 GPa and Poisson ration ϑ=0.26. This approximately corresponds to the room temperature elastic constants of single crystalline Ni-based superalloys. The unit cell (Figure 1a) is discretized in 128×128×128 voxels and contains a square-shaped inclusion discretized in 32×32×1 voxels corresponding to an Eshelby-like square prismatic loop perpendicular to the *z*-axis. In order to generate a shift of the upper surface of the inclusion relative to its lower surface by a Burgers vector $b\left(0,\;0,\;b_{3}\right)$, only the voxels within the inclusion are submitted to a non-zero eigenstrain tensor defined as: $\varepsilon_{ij}^{*}=0$ except $\varepsilon_{33}^{*}=1$. Then, we have $b_{3}=t\times \varepsilon_{33}^{*}$ where t the thickness of the inclusion in the *z*-direction (i.e., the voxel size). This displacement field computed with the FFT algorithm using the different Green operators defined in Section 2.1 is represented along *z*-axis in Figure 1. 

When computed along a line crossing a dislocation loop, the displacement field exhibits a discontinuity with a jump equal to Burgers vector b. This is indeed observed in Figure 1. However, the displacement field computed with the usual Green operator B (Figure 1b) also shows spurious oscillations as soon as the discontinuity is approached. These oscillations (numerical artifacts) are not observed with the periodized operators $B^{\prime }$ and $B^{\prime \prime }$. An artificial damping of the oscillations in Figure 1b (such as a low pass filtering) might smooth these oscillations, but it would also smooth the discontinuity, which is not searched.

### 2.3. Voxelization Effect on the Displacement Field

**Figure 2:**
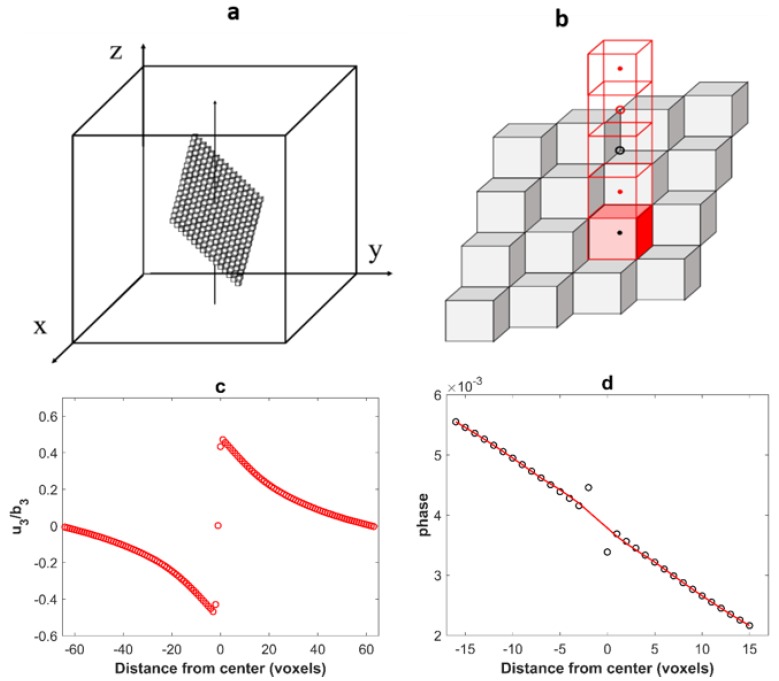
(**a**) Modeling of a dislocation loop in a (111) plane as a layer of voxels with eigenstrain; (**b**) position of the computed points relative to the transformed voxels with eigenstrains; (**c**) plot of the displacement field $u_{3}$ (normalized by $b_{3}$) along the *z* direction for the dislocation loop of Figure 2; (**d**) local oscillation of the phase due to the voxelization of the dislocation loop (the representation is made for 32 voxels centered in the unit cell along *z* direction). The red line is approximately equal to the phase expected for this displacement field.

While the displacement field computed for dislocation loops having their planes parallel to the faces of the simulated volume is correctly given with discrete Green operators $B^{\prime }$ or $B^{\prime \prime }$, voxelization artifacts appear for inclined loops, as shown with $B^{\prime }$ in Figure 2 for a dislocation loop with a $\left[0\bar{1}1\right]$ Burgers vector lying in a (111) slip plane of a fcc crystal. The eigenstrain tensor is constrained in the region occupied by the dislocation loop (transformed voxels) and is given by:

(6) $$\;\varepsilon_{ij}^{*}=\frac{A_{s}}{2V}\left(n_{i}b_{j}+n_{j}b_{i}\right)\;$$

where $A_{s}$ is the area on which planes with normal $n\left(n_{1,}n_{2},n_{3}\right)$ has slipped by a relative amount $b\left(b_{1,}b_{2},b_{3}\right)$ and V is the volume occupied by the dislocation loop [40,41]. As before, the dislocation loop is 32 voxels wide in the *x* and *y* directions, and 1 voxel thick but now with a z position such that x+y+z=constant. The displacement has been computed at the center of voxels with the periodized operator $B^{\prime }$ along *z* (Figure 2b). As in Figure 1c, the displacement in the center of a voxel belonging to the loop plane (black dot in the reddish transformed voxel in Figure 2b) is zero. The displacement in the first neighboring voxels (red dot in Figure 2b) are shifted relative to the expected position, so that the displacement difference between these voxels is significantly lower than ***b***, see Figure 2c. It can be checked in Figure 2b that each of these voxels shares three faces with a transformed voxel. A more detailed analysis shows that the second neighbors (which share three edges with transformed voxels) are also slightly shifted in the opposite direction. The result is shown in Figure 2d with: a strong localized oscillation of the phase (taken here as the displacement modulo ***b***).

Although the amplitude of this shift is small (less than 10% of the Burgers vector) it has unwanted consequences on the diffraction peak simulation:

The dislocation loops are surrounded by four impaired layers of voxels: As the scattered X-ray amplitude is proportional to the Fourier transform of ***G****·**u*** (see Equation (8) in Section 4), we can expect a phantom streak in the intensity in a direction perpendicular to the loop plane.

The displacement field near the edges of the loop (near the dislocation line) will be quite different from its expected value, and the strain field will not vary with the distance r to the dislocation line as 1/r. This will strongly affect the tails of the diffraction peaks.

## 3. Sub-Voxelization Method to Correct Voxelization Artifacts

### 3.1. Sub-Voxelization Method

**Figure 3:**
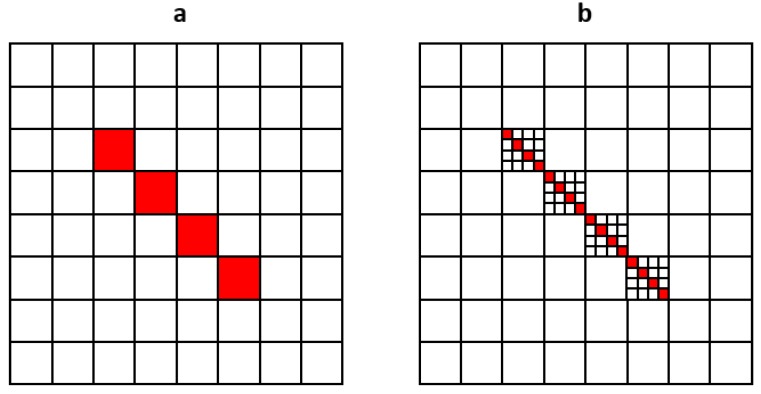
2D representation of a dislocation loop in a tilted plane on a (8×8) fast Fourier transform (FFT) grid: (**a**) with a homogeneous eigenstrain $\varepsilon^{*}$ in the voxels occupied by the dislocation loop; (**b**) with each voxel subdivided into 4 × 4 sub-voxels, only 4 of which have a $4\;\varepsilon^{*}$ eigenstrain field.

The (conceptually) simplest way to remove this voxelization artifact would be to work on a multiple grid (to multiply the number of voxels along each direction by 2, 4, or more), then to downsample the displacement field data. In that case, FFT algorithms would lose much of their interest due to these more demanding computational efforts. We show below that this can be done in a more “economical”—and simple—way by applying a patch to the FFT-computed displacement field. The basic method is to compute, on the same grid, the difference vector:

(7) $$\;\Delta_{i}\left(x\right)=u_{i}^{sub}\left(x\right)-u_{i}^{hom}\left(x\right)\;$$

where $u_{i}^{sub}\left(x\right)$ is the displacement vector calculated for voxels where this eigenstrain is concentrated on a single plane of sub voxels (Figure 3b) and $u_{i}^{hom}$ the displacement field in direction *i* of voxels with a uniform eigenstrain (Figure 3a). For the sake of clarity, we use 2D diagrams in Figure 3, but here the technique is applied to real 3D problems.

**Figure 4:**
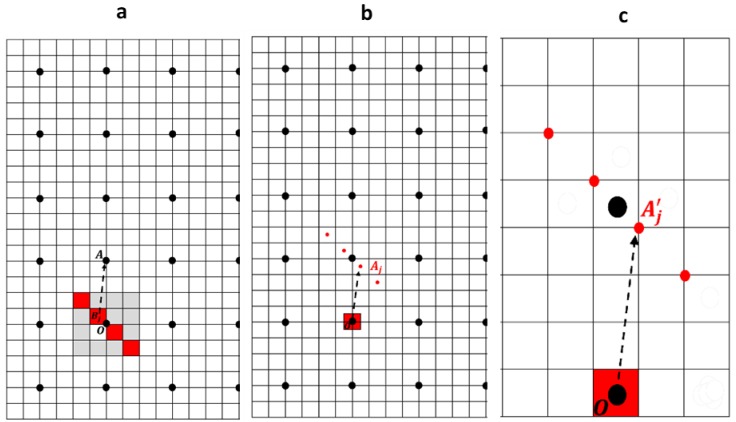
(**a**) 2D representation of the computational grid. The black dots correspond to the voxels centers. A voxel with center O is discretized in 4×4 in 2D (4×4×4 in 3D) sub-voxels. The red sub-voxels have a non-zero eigenstrain. We want to compute the displacement field at point A, due to these deformed sub-voxels centered at $B_{j}$. (**b**) Displacement field generated by a deformed sub-voxel centered at O on a row of sub-voxels centered at $B_{j}$ such as $OA_{j}$ =$\;B_{j}A$. The sum of these displacements is equal to the previous displacement field. (**c**) Displacement field generated by a deformed voxel centered at O on a row of voxels (computed at the corners $A{{}^{\prime }}_{j}$ using Green operator $B^{\prime \prime }$) such as $OA{{}^{\prime }}_{j}$ =$\;nB_{j}A$. This sum is equal to the previous sum.

In order to compute the displacement due to sub-voxels, we use a N×N (N×N×N) grid for 2D (resp. 3D) problems where each voxel can be subdivided into n×n (n×n×n) sub voxels. Only *n* (n×n) sub voxels are submitted to an eigenstrain field. At a point A of the grid (black dots, Figure 4a), we need to compute the sum of the displacements $u_{i}^{j}$ due to the *n* (n×n) sub voxels *j* (center $B_{j}$) within a voxel centered at point O. This sum is equivalent to the sum of the displacements due to a strained sub voxel at point O on the grid points $A_{j}$ such as $OA_{j}$ =$\;B_{j}A$ (Figure 4b). It is also equivalent to the sum of the displacements $u{{}^{\prime }}_{i}^{j}$ due to a *full* voxel at point O on the initial grid on points $A{{}^{\prime }}_{j}$ such as $OA{{}^{\prime }}_{j}$ =$\;nB_{j}A$ (Figure 4c). The only difference between these last two sums is due to the long-range strain field, and approximately results in a linear drift of the displacement. As the end of the vectors $OA{{}^{\prime }}_{j}$ does not lie on the grid points (voxel centers) but on the corners of the voxels, the $u{{}^{\prime }}_{i}^{j}$ displacements must be calculated with the shifted operator B″ (Equation (5)). A last point is the scaling of the $u_{i}^{j}$ and $u{{}^{\prime }}_{i}^{j}$ sums during the operations of Figure 4. To keep the one Burgers vector jump between both sides of the sub voxels plane in Figure 4a, the eigenstrain in the sub voxels must be multiplied by *n*. The backwards change of scale requires a division by *n*: there is no scaling factor between $u_{i}^{hom}$ and $u_{i}^{sub}=\sum u{{}^{\prime }}_{i}^{j}$.

We need to compute $\Delta_{ij\_pl}\left(x\right)$ the difference in displacement in direction *i* due to a voxel which belongs to the plane “pl” (for fcc “pl” is equal to $\left(111\right),\left(\bar{1}11\right),\left(1\bar{1}1\right),\left(11\bar{1}\right)$) of a dislocation loop with a Burgers vector *j* at a position x relative to the transformed voxel. In practice, in a material with cubic symmetry, it is sufficient to compute $\Delta_{13\_\left(111\right)}\left(x\right)$ and $\Delta_{33\_\left(111\right)}\left(x\right)$, and to use the symmetries of the cube (fourfold [001] axis, threefold [111] axis, and $\left(1\bar{1}0\right)$ symmetry plane) (and suitable exchanges of the components of x) to obtain the required components. As can be seen in Figure 2b, $\Delta_{ij\_pl}\left(x\right)$ is non-zero only for the neighbors of the transformed voxel, except the drift due to the long range strain alluded above. The final computational procedure to determine $\Delta_{ij\_pl}\left(x\right)$ and use the patch is now detailed:

Compute the field $c^{0}:\varepsilon^{*}$ defined in Equation (1) for an isolated voxel with the eigenstrain associated to a dislocation loop (Equation (6)) with a Burgers vector [001] in a (111) plane (see Figure 2).

Compute the displacement field in directions *x* ($u_{1}^{hom}$) and *z* ($u_{3}^{hom}$) at the voxels’ center around the transformed voxel by convolution with the discrete periodized operator $B{}^{\prime }$ (Equation (4)).

Compute the displacement field in directions *x* and *z* at the voxels’ corners around the transformed voxel by convolution with the shifted operator $B^{\prime \prime }$ (Equation (5)).

Calculate the $u_{1}^{sub}=\sum u{{}^{\prime }}_{1}^{j}$ and $u_{3}^{sub}=\sum u{{}^{\prime }}_{3}^{j}$ sums (n×n terms for each sum) as in Figure 4c, then the raw $\Delta_{13\_\left(111\right)}\left(x\right)$ and $\Delta_{33\_\left(111\right)}\left(x\right)$ for $\left(x_{1},x_{2},x_{3}\right)$ going from −3 to 3 times the voxel size *t*.

Use the farthest voxels to correct the drift of the components so that all terms for large x are zero, and keep non zero only the terms for the first three neighbors.

The patch can then be applied on the raw (FFT-based) displacement field by adding the convolution of all transformed voxels of the different slip systems by the relevant $\Delta_{ij\_pl}\left(x\right)$.

### 3.2. Results

For numerical tests, we used the same 128×128×128 grid as before, and the transformed voxel was divided into 8 × 8 × 8 sub voxels (using a reference medium with the same elastic constants as before). Only the final values in units of b (after drift correction) of $\Delta_{13\_\left(111\right)}\left(x\right)$ and $\Delta_{33\_\left(111\right)}\left(x\right)$ are used and other components are obtained by symmetries (Appendix A).

**Figure 5:**
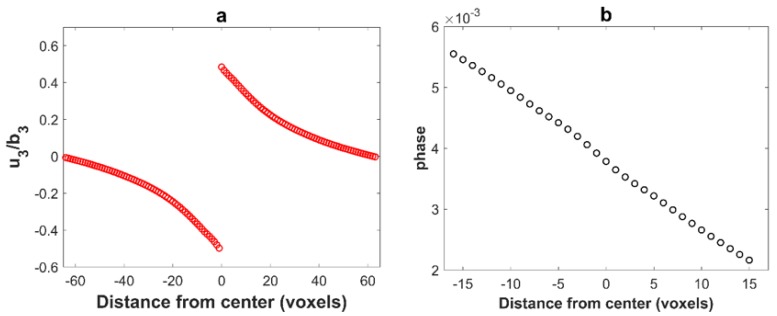
(**a**) Plot of the displacement field $u_{3}$ (normalized by $b_{3}$) along the *z* direction for dislocation loop illustrated on Figure 2. The voxelization artifacts are removed by the sub-voxel method described above. (**b**) The phase (i.e., the displacement modulo a Burgers vector). With this correction, the phase is almost continuous.

The patch was used on the same configuration as in Figure 2. Figure 5a shows the resulting displacement field and Figure 5b the phase (i.e., the displacement modulo a Burgers vector) in Burgers vector units. As it can be observed from Figure 5a, the voxelization artifacts of the displacement field are removed thanks to the sub-voxelization method. In addition, the resulting phase varies smoothly even during the crossing of the dislocation loop which is more realistic, see Figure 5b.

## 4. Application on Diffraction Peak Simulation

**Figure 6:**
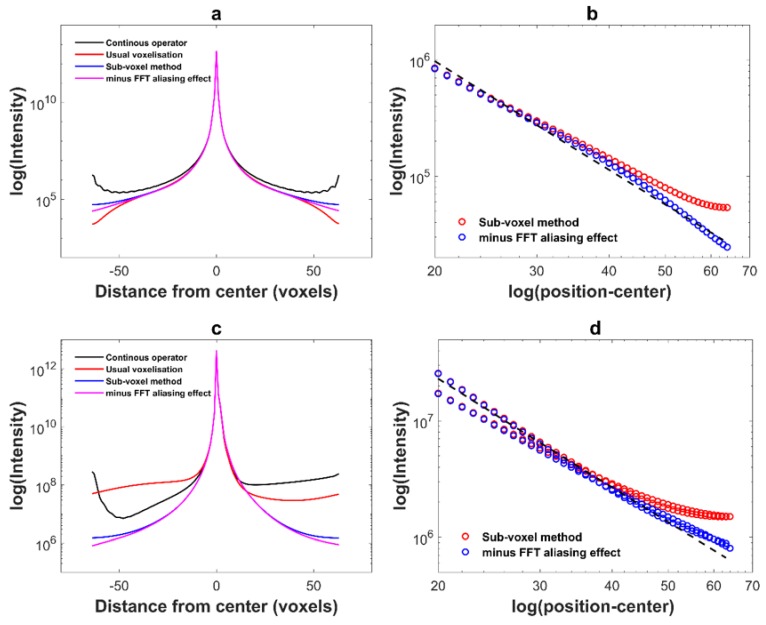
Simulated diffracted intensity as a function of the pixel position (logarithmic scale). 3D configuration is represented in a 1D plot by making the sum in each plane along an *x*-axis. Different way for computing the displacement fields are studied for a dislocation loop with a $\frac{a}{2}\left[0\bar{1}1\right]$ Burgers vector lying in a (111) slip plane. (**a**) Diffracted vector studied is ***G*** (200) corresponding to ***G***·***b*** = 0. (**b**) Log/log representation of the intensity vs. $\left|i-i_{0}\right|$. (**c**) and (**d**) same as (**a**) et (**b**) but the studied diffracted vector is ***G*** (002) (***G***·***b*** = 1).

In this section, we show simulated diffraction peaks in order to point the effects of voxelization artifacts and of the patch on numerical results. Under kinematical conditions and assuming a coherent beam, the amplitude of a diffracted wave at a position q in the vicinity of a reciprocal G lattice vector is [14,18,42,43,44]:

(8) $$\;A\left(q\right)=FT\left[A_{0}\left(x\right)\times F\left(G,x\right)\times exp\left(-2i\pi \;G\cdot u\left(x\right)\right)\right]\;$$

where x is the position of the scattering atom, $A_{0}\left(x\right)$ is the amplitude of the incidence wave, F(G,x) is the local structure factor, and u(x) the displacement field. The scattered intensity is I(q) = ${\left|A\left(q\right)\right|}^{2}$. For a face-centered cubic crystal, this intensity is non zero when ***G*** (*h*, *k*, *l*) is such as *h*, *k*, and *l* have the same parity. Here two diffraction vectors ***G*** (200) and ***G*** (002) are used. They respectively correspond to ***G***·***b*** = 0 and ***G***·***b*** = 1. The 3D diffracted intensity has been calculated using the FFT instead of the continuous Fourier transform, then summed in the planes perpendicular to the ***G*** vector to obtain a linear plot along ***G*** equivalent to a *I*(2*θ*) plot. In Figure 6a (***G*** (200)) and Figure 6c (***G***(002)), we show the diffracted intensity (logarithmic scale) as a function of the pixel position i, and in Figure 6b,d a logarithmic/logarithmic plot of the intensity vs. $\left|i-i_{0}\right|$ where $i_{0}$ is the center of the peak. In order to only study the effect of the displacement fields, we set $A_{0}\left(x\right)=1$ and F(G,x)=1 for these simulations.

The peak shape near the top of the peaks is the same for both computing methods. It is perfectly symmetric in the ***G***·***b*** = 0 case and exhibits a bump on the right side for ***G***·***b*** = 1. The long-range behavior is, however, quite different. When the displacement field has been calculated with the usual truncated operator (black line), a phantom peak is observed at large $\left|i-i_{0}\right|$ (at large *q*), which is due to the short period oscillations near the displacement field discontinuity (Figure 1a). The behavior of the peak calculated with the modified Green operator (red curve) is only slightly better: the intensity at large q is underestimated in one case and overestimated in the other. When the intensity has been calculated with the sub voxel patch (dark blue curve) the long range intensity follows the expected $I_{0}{\left|i-i_{0}\right|}^{-3}$ law [45,46]: the peak tails are indeed related to the highly distorted zones near the dislocations’ cores. However, the dark blue curve saturates at very large ***q***. We suppose this is due to the use of the FFT instead of the continuous Fourier transform in the calculation of the scattered amplitude (Equation (8)). The plot of Figure 6 represents only one period in Fourier space, and is repeated over and over on all Fourier space. We can now calculate the intensity of the tails of these repetitions:

(9) $$\;I_{neib.}=\sum I_{0}{\left|i-i_{0}-128m\right|}^{-3}\;$$

where m varies from −5 to 5 (zero excluded). If we now plot the difference between the dark blue intensity curve and this background line, we obtain the pink curve. On the log./log. plots, Figure 6b,d, it can be checked that this curve follows the $I_{0}{\left|i-i_{0}\right|}^{-3}$ law to the end. Thus, the residual error in the intensity computed by FFT results of the FFT itself, and not from an error on the sub voxel-corrected displacement field. If the number of voxels is increased to 512^3^ or 1024^3^ while keeping the physical size of the representative volume constant, this residual error should fall down to undetectable levels.

## 5. Conclusions

In this paper, we have shown that although the use of a periodized Green operator in the FFT-based method improves the final displacement field solution in a representative volume containing discontinuities (dislocation loops), artifacts due to the voxelization of the dislocation loop planes are still present with respect to analytical solutions. These artifacts have unwanted consequences on the tails of diffraction peaks simulated by using this displacement field as input data.

We have introduced a patch through a sub-voxelization method, which corrects these artifacts by simulating the displacement field without employing a finer grid resolution. A simple construction method for this patch has been given and the patch can be used in a single post-processing step to modify the initial FFT-based displacement field.

The modified displacement field has been used to simulate one-dimensional diffraction peaks. The procedure strongly improves the shape of the peaks’ tails, i.e., it gives a good description of the displacement field and the phase near the dislocation lines.

## Appendix A

$\Delta_{33\_\left(111\right)}\left(x_{1},x_{2},x_{3}\right)$ and $\Delta_{13\_\left(111\right)}\left(x_{1},x_{2},x_{3}\right)\;$ have these symmetries due to the permutation properties of the plane (111):

$$\;\Delta_{33\_\left(111\right)}\left(x_{1},x_{2},x_{3}\right)=\Delta_{33\_\left(111\right)}\left(x_{2},x_{1},x_{3}\right)\;$$

$$\;\Delta_{33\_\left(111\right)}\left(x_{1},x_{2},x_{3}\right)=-\Delta_{33\_\left(111\right)}\left(-x_{1},-x_{2},-x_{3}\right)\;$$

$$\;\Delta_{13\_\left(111\right)}\left(x_{1},x_{2},x_{3}\right)=-\Delta_{13\_\left(111\right)}\left(-x_{1},-x_{2},-x_{3}\right)\;$$

The others values of $\Delta_{ij\_\left(111\right)}\left(x\right)$ are given as function of $\Delta_{33\_\left(111\right)}\left(x_{1},x_{2},x_{3}\right)$ and $\Delta_{13\_\left(111\right)}\left(x_{1},x_{2},x_{3}\right)$:

$$\Delta_{11\_\left(111\right)}\left(x_{1},x_{2},x_{3}\right)=\Delta_{33\_\left(111\right)}\left(x_{3},x_{2},x_{1}\right)\;\Delta_{12\_\left(111\right)}\left(x_{1},x_{2},x_{3}\right)=\Delta_{13\_\left(111\right)}\left(x_{1},x_{3},x_{3}\right)\;$$

$$\Delta_{22\_\left(111\right)}\left(x_{1},x_{2},x_{3}\right)=\Delta_{33\_\left(111\right)}\left(x_{1},x_{3},x_{2}\right)\;\Delta_{31\_\left(111\right)}\left(x_{1},x_{2},x_{3}\right)=\Delta_{13\_\left(111\right)}\left(x_{3},x_{2},x_{1}\right).\;$$

$$\Delta_{32\_\left(111\right)}\left(x_{1},x_{2},x_{3}\right)=\Delta_{13\_\left(111\right)}\left(x_{3},x_{1},x_{2}\right)\;\Delta_{21\_\left(111\right)}\left(x_{1},x_{2},x_{3}\right)=\Delta_{13\_\left(111\right)}\left(x_{1},x_{3},x_{2}\right)\;$$

$$\;\Delta_{23\_\left(111\right)}\left(x_{1},x_{2},x_{3}\right)=\Delta_{13\_\left(111\right)}\left(x_{2},x_{1},x_{3}\right)\;$$

The value of $\Delta_{33}\left(x_{1},x_{2},x_{3}\right)$ and $\Delta_{13}\left(x_{1},x_{2},x_{3}\right)$ for the remaining plane $\left(\bar{1}11\right),\left(1\bar{1}1\right),\left(11\bar{1}\right)$ are obtained using these symmetries:

$$\;\Delta_{33\_\left(\bar{1}11\right)}\left(x_{1},x_{2},x_{3}\right)=\Delta_{33\_\left(111\right)}\left(x_{2},-x_{1},x_{3}\right)\;\;\Delta_{13\_\left(\bar{1}11\right)}\left(x_{1},x_{2},x_{3}\right)=\Delta_{13\_\left(111\right)}\left(-x_{1},x_{2},x_{3}\right)\;\;\Delta_{33\_\left(1\bar{1}1\right)}\left(x_{1},x_{2},x_{3}\right)=\Delta_{33\_\left(111\right)}\left(-x_{2},x_{1},x_{3}\right)\;\;\Delta_{13\_\left(1\bar{1}1\right)}\left(x_{1},x_{2},x_{3}\right)=\Delta_{13\_\left(111\right)}\left(x_{1},-x_{2},x_{3}\right)\;\;\Delta_{33\_\left(11\bar{1}\right)}\left(x_{1},x_{2},x_{3}\right)=-\Delta_{33\_\left(111\right)}\left(x_{2},x_{1},-x_{3}\right)\;\;\Delta_{13\_\left(11\bar{1}\right)}\left(x_{1},x_{2},x_{3}\right)=-\Delta_{13\_\left(111\right)}\left(x_{1},x_{2},-x_{3}\right)\;$$
